# “Trust in God, but tie your donkey”: Holy water priest healers’ views on collaboration with biomedical mental health services in Addis Ababa, Ethiopia

**DOI:** 10.1177/13634615241227681

**Published:** 2024-02-05

**Authors:** Yonas Baheretibeb, Dawit Wondimagegn, Samuel Law

**Affiliations:** 1 37602 Addis Ababa University; 2 7938 University of Toronto

**Keywords:** Ethiopia, religious healer, holy water, collaboration, mental illness

## Abstract

This exploratory qualitative study examines holy water priest healers’ explanatory models and general treatment approaches toward mental illness, and their views and reflections on a collaborative project between them and biomedical practitioners. The study took place at two holy water treatment sites in Addis Ababa, Ethiopia. Twelve semi-structured interviews with holy water priest healers found eight notable themes: they held multiple explanatory models of illness, dominated by religious and spiritual understanding; they emphasized spiritual healing and empathic understanding in treatment, and also embraced biomedicine as part of an eclectic healing model; they perceived biomedical practitioners’ humility and respect as key to their positive views on the collaboration; they valued recognition of their current role and contribution in providing mental healthcare; they recognized and appreciated the biomedical clinic's effectiveness in treating violent and aggressive patients; they endorsed the collaboration and helped to overcome patient and family reluctance to the use of biomedicine; they lamented the lack of spiritual healing in biomedical treatment; and they had a number of dissatisfactions and concerns, particularly the one-way referral from religious healers to the biomedical clinic. The study results show diversity in the religious healers’ etiological understanding, treatment approaches and generally positive attitude and views on the collaboration. We present insights and explorations of factors affecting this rare, but much needed collaboration between traditional healers and biomedical services, and potential ways to improve it are discussed.

## Introduction

Traditional healers in Africa work closely with prevalent and culturally ingrained explanatory models of mental illness, often involving religious, spiritual, mythological and folklore accounts (Abbo, 2011; Mirza et al., 2006; Patel et al., 1995; Sorsdahl et al., 2009, 2010a, 2010b). In Ethiopia, the most common traditional healing involves holy water treatment, predicated on the dominant explanatory model that evil spirit possessions or interferences are responsible for patients’ mental illnesses (Alem et al., 1999; Bekele et al., 2009; Girma & Tesfaye, 2011; Selamu et al., 2015; Teferra & Shibre, 2012). Studies have found that more Ethiopians prefer and value the services of traditional healers, and other forms of informal help, such as those from family and friends, over those of the biomedical health centers for their major illnesses, and particularly for the treatment of mental disorders (Shibre et al., 2008; Shumet et al., 2021).

Improving access to mental health services for all is an important step to improve global population health (World Health Organization [WHO], 2001), particularly in developing economies. The WHO has emphasized engaging culturally competent providers who are versed in patients’ belief systems and explanatory models to improve access to care (WHO, 2008). Traditional healers also fit this description and play an indispensable role in mental healthcare globally (Ae-Ngibise et al., 2010; Cooper, 2016; Ndetei, 2007; Patel, 2011; Pham et al., 2021). Two WHO strategies endorse the involvement of traditional healers: the Traditional and Complementary Medicine Strategy 2014–2023, which aims to achieve universal health coverage (WHO, 2013a); and the Mental Health Action Plan 2013–2020, which promotes mental well-being (WHO, 2013b).

However, the relationship between the traditional and biomedical forms of mental healthcare has historically been fraught (Calabrese, 2013; Mbwayo et al., 2013). There are different etiological understandings, entrenched distrust and unfavorable regard for each other, not least in that biomedical practitioners often see traditional healers as unscientific, irrational and superstitious (Akol et al., 2018; Incayawar et al., 2009). Not surprisingly, there is a dearth of collaborations between the two systems (Keikelame & Swartz, 2015; Musyimi et al., 2016; Robertson, 2006; Vaka et al., 2009). The limited, but encouraging reports of formal and informal collaboration tend to focus on gaining traditional healers’ trust and facilitating their referral to biomedicine, or on getting them to use biomedical knowledge (Joint United Nations Programme, 2006). More active collaborations that are based on respectful, equitable and cooperative relationships are called for (Campbell-Hall et al., 2010; Pouchly, 2012; Solera-Deuchar et al., 2020).

In Ethiopia, informal links between traditional religious healers and biomedical professionals were first documented in a 1968 ethnographic study of the Ghion holy water site, where Giel and colleagues remarked that “[the priest] sends some of his cases to the nearby health centre, and there is a two-way trickle of patients between his place and Amanuel Mental Hospital in Addis Ababa” (p.64) (Giel et al., 1968). More recently, the late Ethiopian Orthodox Church Patriarch His Holiness Abune Paulos was known to endorse and promote collaboration: “Both the Holy Water and the medicine are gifts of God. They neither contradict nor resist each other” (Tadesse, 2007). Currently, there is a collaborative project forged between biomedical practitioners from the Ethiopian Mental Health Society (an non-governmental organization [NGO]), the Department of Psychiatry at Addis Ababa University, and St Michael's and St Mary's churches (two closely located holy water treatment sites) in Addis Ababa, Ethiopia (Baheretibeb et al., 2021).

We aimed to study this collaborative project by examining the religious healers’ explanatory models of mental illness, general treatment approaches, and their perception, attitude and willingness regarding cross-system collaboration. We hope to gain understanding in this area to inform further strategies to collaborate and improve access to mental health services in Ethiopia.

## Methods

### Brief introduction to Ethiopian traditional healing concepts and approaches

Although not systematically researched and with no consistent diagnostic criteria available, some examples of culturally recognized conditions or syndromes related to mental illness are illustrative. In the Amharic language, “*cherqun yetale*” denotes a severely mentally ill person unfit for any responsibility; “*wofeffe*” or “*nik*” implies that a person is not too reliable because he/she has inconsistent behavior due to episodic explosiveness or other unusual changes in his/her interpersonal communication; “*abbsho*” describes an individual who had taken some psychoactive drug (usually herbs) at some earlier point in his/her life, and later develops psychoses-like behavior whenever taking an alcoholic drink (Alem et al., 1995).

There are at least three different types of spirits involved in mental illness: (1) the “*Zar*”, considered to run in the family lineage; (2) the “*Ayine Tila*”, which are considered to be luck spoilers and bring misfortune and obstacle to one's life; and (3) the “*Buda*” or “evil eye”. People with mental health problems are thought to be cursed or possessed by one or more of these specific spirits. Reasons for the affliction may be related to transgressions of God's rule or ignorance of God's guidance, offenses in matters of wealth or temptations like sex, or people themselves calling on the evil spirits in bad faith, and so on. The possessed individual’s symptoms and intensity will depend on the specific possessing spirit(s). Some typical attributed manifestations include hearing voices, talking to oneself, laughing alone, unusual shouting or singing, unexplained fainting, a refusal to eat or drink, and suicidal thoughts. Treatment often involves holy water (e.g., splashing or bathing in holy water), and other forms such as exorcism, the process of driving out evil spirits by means of prayers, anointment with oil, and the use of “*Emenet*” or holy ash).

### Collaborative project background

The project began in 2012 with several small meetings between the first author (YB) and the religious healer leadership that led to a series of more formal workshops at each other's locations of practice—at the churches, and on campus at Addis Ababa University and a major general hospital. The workshops engaged over 100 priests and healing attendants, with the aim of gaining mutual understanding, trust and levels of comfort among the participants.

The highlights of the workshops included learning about each other's theoretical bases and explanatory models of illness, sharing case scenarios, and discussions on difficult issues encountered in both practice settings, such as stigma, discrimination, maltreatment and use of physical restraints to manage challenging patients. These discussions helped to forge a sense of shared purpose and a goal to improve patient care. A concrete agreement resulting from these collaborative engagements was the creation of a biomedical outpatient clinic, on-site in the holy water treatment community. The mandate was to welcome any patient seeking holy water treatment at the site who was also interested in receiving biomedical consultations and possible treatment through drop-ins or referrals. The clinic started in 2012, and referrals from priests and attendants came in within 2 months of opening. The clinic is staffed by western-trained local Ethiopian psychiatrists and senior residents. The services are free of charge, provided once every 2 weeks, for 6–7 h each time (Baheretibeb et al., 2021).

### Study overview

The main research questions were: what are the religious healers’ explanatory models of mental illness; and what are their perceptions, attitude, willingness, experiences and reflections from collaborating with the biomedical model? Our approach was broadly inspired by Kleinman's explanatory model and framework (Kleinman, 1980), with attention to the interaction and collaboration between health sectors (Kleinman, 1978), and informed by Engel's biopsychosocial concept (Engel, 1977; 1978), with an added spiritual dimension (Katerndahl, 2008; Sulmasy, 2002). Our specific research methodology was informed by constructivist grounded theory—a systematic qualitative research approach that emphasizes the generation of in-depth, nuanced, contextual and sensitive understanding rooted in data, assumes a relativist approach, acknowledging multiple perspectives and realities of both the researchers and study participants, and takes a reflexive stance toward observed and reported data, while allowing the researchers to carry out a literature review on the general subject to form preliminary hypotheses to inform the research (Charmaz, 1990, 2014; Silverman & Marvasti, 2008).

We developed a detailed semi-structured interview that aimed to explore the stated research questions. A select sample of the interview questions (all contained “probe as needed” instructions) are:

- Can you tell me what you think caused mental health problems you see in patients at holy water sites?
- What do people in your church, friends or others in your community think caused the [problem]?
- How do you think the problem of mental illness affects the patients at holy water sites? Their body? Their mind? Their spiritual well-being?
- What do you think is the best way to deal with this kind of problem?
- What are your opinions and views of the collaborative project with biomedical services?

The interview guide was translated into *Amharic,* the most common local language.

### Study settings

The study setting broadly involved the holy water treatment community based at St Michael's and St Mary's churches in Entoto, Addis Ababa and comprised ∼1000 individuals, including patients, their attendants, some family members, and the priests and assistants. The patients reside in simple dwellings near the two churches. Both church sites have a natural supply of spring water that has been considered holy through church recognition and historically recorded healing miracles. At St Michael's, for example, the miracle involved a severely mentally ill man—who appeared to suffer from a manic-depressive illness by western biomedical interpretations—taking the waters and recovering. In addition to holy water, the priests also offer various forms of exorcism, and religiously based counseling (based on Orthodox Christian beliefs and concepts, focusing on spiritual concerns and everyday life struggles). The holy water services are provided free of charge.

### Participants and recruitment

We employed a purposive sampling approach to recruit priests at the two churches who attended the consultation workshops and were identified by the church leadership as working with mentally ill patients. All priests who were approached accepted the invitation to participate. The final number of participants was 12 (see Table 1 for details).

**Table 1.** Basic demographic information of participants.

Total no. of participants	12
Age (years)	Range: 37 to 78; mean: 57.5
Gender	All male
Place of practice	5 at St Mary's; 7 at St Michael's
Work experience (years)	Range: 12 to 43; median: 35
Additional education background	4 of the 12 also had a modern western education
Spiritual healing abilities	2 of the 12 healers were diviners (with the ability to carry out full exorcism, a role reflecting more experience, because exorcism involves a human medium and only a diviner has the privilege of baptizing a patient and serving as a medium).
	The other 10 were *Atmaqi*, or holy water priests, who has the ordained power to transform ordinary water into *tsebel* (holy water) by chanting Scripture and using a special cross

### Data collection

In March 2020, we conducted 12 single-session interviews, each lasting about 45–60 min, held at a private location convenient to the participant; interviews were tape-recorded and supplemented with field notes. The interviews were coordinated and conducted in the local language (*Amharic*) by a research assistant (RA) who has a sociology background, experiences in qualitative research, *Amharic* proficiency and previous work experience at a holy water site. The participants were informed that their responses would be recorded but no personal information would be identified in the analysis or discussion to optimize privacy and candidness.

### Data analysis

Interviews were transcribed from *Amharic* into English by the same RA who conducted the interviews. The RA checked the accuracy of the transcripts while taking care to preserve important local language concepts and nuances. A selection of the English translations was compared against the original *Amharic* transcripts by YB (the first author, a psychiatrist with broad clinical and mental health system experiences).

The research data were analyzed using a thematic analysis approach with coding for the salient materials, and comparing statements, emotions and responses within and between participants (Clarke & Braun, 2014). The basic “sensitizing” concepts were used to inform initial thematic coding, while broader and new content and concepts were actively captured (Bowen, 2006). The RA and the first author independently coded and made memos and generated categories and themes on 8 of the 12 English transcripts. The coding and memos from both were reviewed and compared and then consolidated to enhance the completeness and credibility of the analyses. Any significant difference—mostly minor—was reviewed and discussed, and resolved through reaching a consensus. Notes on the discussions were also taken to inform the ongoing coding process. As analysis proceeded, earlier transcripts were re-read and re-coded as necessary to allow re-capturing of newly emerging themes and concepts. Frequent and salient codes were identified, and thematically linked codes were clustered to generate distinct and informative thematic categories. The remaining four English transcripts were then coded by the first author using the final, agreed-upon coding framework. Data saturation was determined by the RA and PI (Guest et al., 2006). Quotations were selected to represent themes in the data.

### Ethical approval

For each interview, the RA contacted the selected potential interviewee in person at a convenient time, explained the purpose of the study, answered any questions, explained the consent process, and finally gained permission to proceed. The study followed all ethical guidelines for research involving human subjects and obtained full written consent for interviews and recordings. The study received approval from the Ethics Board of the School of Medicine, Addis Ababa University.

## Results

### Views on illness explanatory models and treatment approaches

#### Holy water priest healers hold multiple explanatory models of illness, dominated by religious and spiritual understanding

All the priest participants recognized “mental illness” as a distinct category of illness. They described different explanations of illness based on religious epistemologies, and their experiences, understanding and knowledge. They reported multiple explanations on the causes of mental illness—the most prevalent were in references to the Bible, demonic possession, evil spirit witchcraft and bewitchment.

One participant (P12) reported that the cause of mental illness is clearly written in Scripture:

Patients have suffered from mental disorders since the dawn of history. There is an indication of this in the book of Deuteronomy that talks of God's punishment for those who violate divine commands: “The Lord shall smite thee with madness, and blindness, and astonishment of heart.” Deuteronomy 28:28

The same participant also reported:

Another good example was the case of the biblical King Saul, the first King of Israel, who ruled the country 3000 years ago. An evil spirit from the Lord troubled him and evaluation of the passages referring to King Saul's disturbed behavior indicates that he was afflicted by a mental disorder.

One participant (P5) recounted how young girls were easily attacked by hostile glares or evil eye:

If a beautiful young girl is looked at by someone with a bad eye, “Buda,” the girl might show signs of mental illness like fainting, shouting day and night, poor sleep, and fearfulness.

Another participant (P1) reported sprit possession as the cause of mental illness:

In our holy water center many patients present after they have been possessed by “Zar” spirit. Usually I identify the possessed person by physical symptoms that include headaches, restlessness, refusal to eat or communicate, shouting or infertility to your surprise.

One participant (P7) invoked other spirits or spiritual creatures:

In my opinion, most people with mental illness … were attacked by Galen or “Jinn.” Their use of incantation, sorcery, magic or certain rituals might be the cause of mental illness and the only treatment is exorcism—a procedure that involves conducting special ceremonies, burning incense, praying, using holy water, and advice to put amulets containing a written script.

Other participants believed being cursed by the elderly might cause mental illness, as one (P12) said:

For me in my 15 years’ holy water service experience, I learned that one could be cursed for striking parents, swearing at elderly people, stealing livestock, and/or for murder. The consequences of the curse might be childlessness, joblessness, persistent sickness, severe mental disorder and deaths, among others….

Some participants mentioned bewitchment, particularly related to retaliation of an offense or misdeed, as one (P1) described:

If someone takes [steals] someone else's money or wife or material, the person who lost the money or wife or material may do something in retaliation which makes the other person mad. He may do some witchcraft on him which may make him mad.

Beyond the above, the priests also reported excessive alcohol use, *khat* use, drug abuse, heredity, physical illnesses, injury and old age in their explanation. For example, one (P2) such explanation combined the spiritual and physical:

When an individual behaves abnormally we call it mental illness. This might have been disability from the birth or acquired as one is growing up or through evil spirit and demonic power. It can also be as a result of heavy (*khat*) and drug use or head injury causing brain damage. Poor childhood upbringing might cause serious mental illness. Examples include epilepsy, drooling, unexplained silence, talking aimlessly or hitting people and madness. The types include laughing and talking aimlessly, refusal to eat, physical and verbal aggression, harm to self and others, fainting.

Some participants linked physical illness to mental illness, as one (P10) described:

People with severe physical illness like HIV, malaria or nerve illness, if they do not get treatment on time, they might lose their mind and became crazy, or develop self-harm behaviors.

Other participants also described socioeconomic problems and psychological distress related to trauma, migration, sexual abuse, trauma, humiliation, shame and poverty as contributing causes. One priest (P6) summarized:

For a poor person life is terrible—mental and physical illness, humiliation, shame. He is afraid of everything; he depends on everyone. No one needs him. He is like garbage that everyone wants to get rid of him…, all these expose a person to any kind of mental illness.

Another participant (P8) brought attention to the plight of migration-related trauma as a cause:

In my day-to-day experience at the holy water sites I witnessed several young girls who were expelled from Arab countries. Before their return, they experienced sexual abuse, detention, theft of migrants’ belongings, rape, beatings, and killings which resulted in severe mental illness.

#### Priest healers emphasized spiritual healing and empathic understanding in treatment, also embracing biomedicine as part of an eclectic healing model

Participants reported holy water sites to be a safe haven for mentally ill people, and patients find spiritual support and care and avoid alienation, as one (P2) stated:

Mentally ill people are coming to us to take the opportunity to exercise their spirituality and show devotion to their religion, such as giving confession and receiving penance through Father confessors. Above all it is a place where they get emotional, physical and spiritual support.

Most participants emphasized that mentally ill patients need compassion and protection, and strongly believed that holy water could cure mental illness. A majority of priests also believed mental disorders needed multiple treatment methods that amalgamated holy water, prayers and advices from clergymen, and western treatment to attain maximum benefits. In this eclectic healing model, as one participant (P1) stated:

In my observation, people with mental illness are the most neglected, abandoned, and segregated, with multiple physical and mental health problems. They need our compassion in multiple areas—we have to feed them, pray for them, listen to them, cure their mental illness with holy water, send them to a [biomedical] doctor for treatment and protect them from any kind of abuse; we need to pray for them. In my opinion we have to use any kind of healing process to help them.

One priest (P4) highlighted his endorsement of a combined holy water and medication approach:

I am positive about taking medication for treatment of mental illness. I have seen people being cured who had been using both [bio]medicine and holy water. God is gracious; He will not be jealous or sorry if someone takes the medicine together with the holy water. It is written in Scripture. God changes times and seasons; He removes kings and sets up kings; He gives wisdom to the wise and knowledge to those who have understanding; thus, He is the source of knowledge including medications for mentally ill people.

Some participants evoked the respected words of the late Ethiopian Orthodox Church Patriarch His Holiness Abune Paulos, who told a large gathering of the faithful: “What we are saying is taking the drugs is neither a sin nor a crime.” For example, one participant (P5) recounted:

It is a religious obligation for me to respect His Holiness’s declaration to take both holy water and medication for people living with HIV/AIDS. This declaration applies to people with mental illness, too. Regarding the controversial question of medication or holy water or both? My answer to this is: there is an Ethiopian saying, “Trust in God, but tie your donkey.” Thus, take both.

One participant (P9) argued further for a combined approach from a biblical perspective:

When we consult Scripture, we find nowhere that God commands Christians to avoid doctors or medicine, nor to refuse blood transfusions, inoculations or surgery. In fact, we often see medical knowledge praised as a gift from God for the benefit of people. For example, in Genesis 17:10–14 the Lord commands the procedure of circumcision to Abraham. God ordained this minor surgery for a very specific, spiritual purpose, but nevertheless this example demonstrates that relying on a medical procedure isn’t contrary to obeying God. In fact, sometimes it's necessary in obeying God.

However, one participant (P6) expressed that his approach was exclusively using spiritual treatment:

I strongly believe that sin and the sin of others around are often the catalyst for creating the circumstances that lead to destructive behaviors, emotional pain, depression, anxiety and other ailments. The Lord's prescription for these situations is that we (or others) humble ourselves, repent for the sin, and commit to living in obedience according to His word. The trauma we experience in the meantime is the instrument of discipline the Lord uses with His children to bring us to that moment of repentance. Thus I strongly advise patients to stick to spiritual treatment only.

### Views on the cross-system collaborative model

#### Perception of biomedical practitioners’ humility and respect key to priest healers’ positive views on the collaboration

Regarding the consultative workshops, the overall engagement process and the collaborative project, participants reported that what they valued most was feeling that they were being treated as equals. They appreciated being actively involved in the initial meetings and being given an important role in the planning process. These positive feelings motivated them to enter, and continue with, the collaboration. One participant (P1) reported:

During the consultative meetings, the doctors were very humble and modest. They were non-confrontational; they just listened to our understanding of mental illness and shared their understanding with respect. They were discussing differences and conflicts in worldviews with respect. Throughout our discussions, the doctors were striving to form a dedicated and caring team. I personally learned many things that will help me to improve my patient-care skills.

Another participant (P7) echoed that:

During the first visit to the holy water, the doctors were very humble, respectful and they treated us as influential religious healers at all times. The workshops were opened and closed with prayers. Above all, during the workshops we were given a chance to teach and facilitate the meeting. We were also invited to the hospital to observe how the doctors manage people with mental illness. All these were great means of building trust while enhancing the credibility and breadth of the collaboration.

#### Recognition of the current role and contribution of religious healers in mental healthcare facilitated collaborative engagement

All participants reported that the biomedical practitioners’ acknowledgment of the important role the holy water priests played in the current mental healthcare system contributed to their feeling positive about the collaboration, as one (P10) said:

The doctors acknowledged that we share religious understanding with people with mental illness, the spiritual cause of mental illness, and offer better spiritual and psychosocial care to the neglected mentally ill people than medical doctors. I feel this gives me much-deserved recognition in the community. It also helped us to trust them in further collaboration.

Having a common goal of patient care and some shared values also helped the collaboration; one participant (P3) provided an example:

I think one of the things that's great about people who come to see priests is that it's confidential and it's the same thing with the mental health profession. You can say anything to them and it's great to be able to do that.

Some participants reported mutual respect and learning in the process of collaboration, as one (P2) noted:

We religious healers have got high enthusiasm to collaborate and learn from western-trained doctors and they have also a lot to learn from us.

#### Priest healers particularly appreciated biomedical clinic's effectiveness in treating violent and aggressive patients

Some religious healers liked referring to the clinic patients who had more physical conditions—particularly soft tissue injuries and fractures, because they could not manage these so well. This also applied to patients with active violent and aggressive behaviors, as a participant (P4) stated:

I think families are desperate for help with a relative who is agitated or aggressive, disturbing the household and often the wider community. Such behavior, widely associated with madness, is most often what propels families to come to holy water with the patient often in shackles. In such [a] state, it is impossible to do the religious rituals but with the help of doctors and medications, in a few days, the patient became calm, and we could continue with our religious rituals. That is very impressive. Now I am aware of the problems that I cannot control effectively. This common interest can yield opportunities for organizing collaboration with them.

One priest healer (P8) also reflected:

In the past I used to treat severely mentally ill, agitated patients with their shackles and it was not a comfortable process. The patient was physically suffering, I also was not happy about it. After the collaboration, I started to refer agitated and acutely mentally sick person to the clinic. After one injection, they do a great job. Agitated patient settled down and they unshackled him.

Another participant (P10) was also impressed:

… I adore using the clinic because it really puts a mentally ill person to sleep well and get calmness … I’m a spiritual healer but I have found out that at the outreach clinic there is a very effective medication that immediately calms the person with mental health disorders, especially when they are violent.

Furthermore, some participants have opined on the respective strengths and weaknesses of each modality of treatment, as one (P8) reported:

In my observation, doctors are very good in managing highly agitated and distractive patients; but mild cases like patients with grief, marital problem, family conflict, fear, headache, multiple physical pain, hopelessness … etc., we manage excellently.

#### Religious healers helped to overcome patient and family reluctance to use biomedicine

Some patients and families were reluctant and resistant to attending the clinic. One participant (P11) described the challenges during the process of referral:

Often times families resist my referral to the clinic. They always feel if they sought such help from a doctor, they think it is interfering with God's will. But I always tell them they should understand that all healing comes from God. Looking to the New Testament, Jesus Christ said, “Those who are well do not need a physician, but those who are sick, do” (Luke 5:31). Again, in Luke 4:23 Jesus quotes the proverb, “Physician, heal yourself,” and applied it to Himself. In no case do we find Christ disapproving of medicines or physicians. In fact, one of the Gospel writers, Luke, was a “beloved physician” himself.

Some participants reported that referring patients to the clinic was not an easy task, as one (P10) explained:

Almost always family members strongly believe that emotional or behavioral issues are the symptoms of a spiritual deficit, which must be addressed through holy water, repentance, godly counsel and a spiritual change in accordance with God's word. Before referring the patient, I always conduct multiple family meetings and discussions. In my discussion, I usually mention this very helpful and convincing verse from the Bible (Isaiah 38:21): we find the prophet prescribing a poultice for Hezekiah's boil. While all healing is directed by God, we see the poultice clearly demonstrates that God uses medical procedures at times as a means of delivering the healing He provides. Here is strong evidence that we may be healed by God though the use of sound medical practice!

#### Religious healers lament the lack of spiritual healing in biomedical treatment

Many religious healers pointed out the limitations and shortcomings of the clinic. Quite consistently, the participants highlighted that the core advantage of their care over biomedical care is through spiritual healing. One participant (P6) expressed:

I think, if the Orthodox Christian turns to psychotropic drugs instead, he may mask the true, underlying spiritual cause for his issue. The patient experiences a behavioral or attitude “improvement,” but the change is artificial, a product of chemicals rather than true spiritual change. Like taking *khat*, or alcohol, the person “feels” better for as long as the drug is in the bloodstream, but the true cause for the problem remains untreated. I believe the Orthodox Christians who rely on these drugs to treat mental illness will not grow spiritually from this trial. They avoid the need to examine their life for sins and take corrective steps.

Another participant (P4) felt similarly:

It is clear for me western doctors cannot cure a mental illness, only help some symptoms to be controlled.

This reservation about the biomedical approach, especially regarding how something essential may be lost in biomedical treatments, remained one of the biggest reported differences between the two paradigms.

#### Some biomedical care aspects caused dissatisfaction and concerns, particularly the one-way referral pattern

Participants were forthcoming with their concerns about the collaborative project. For example, one (P9) complained about the conditional acceptance of patients by the clinic:

Last year during Easter time, from St Michael's holy water site I referred a severely mentally ill patient to the clinic. The patient was covered with holy mud “*Emenet*” and the patient looked muddy. On arrival, the doctors chased the patient away accusing them of being dirty…

This regrettable incident has been very instructive for the clinic in improving their sensitivity and awareness. Another participant (P10) had reservations about the medications:

…there is a very effective medication…; however, if they give the person too much of the medication they change them [in]to [a] zombie which makes it difficult to engage them spiritually.

Religious healers tended to want a more integrated system, and stressed that collaboration needed to be two-directional. Several religious healers lamented that the referrals were one-sided and biomedical practitioners did not refer patients to religious healers or refer patients back to them, as one participant (P4) reported:

There are conditions, especially in the beginning of the illness, where the person may be very agitated or restless or aggressive, and I understand that this is not my area to calm him down. However, after the person has settled, the doctors should tell him to go to us for possible spiritual support, but they do not do that.

Another participant (P9) echoed:

I always send patients with severe mental illness to them for management, but they do not give feedback on the referral. I am sure some of the patients under their care need special spiritual support too … we need to get them back.

## Discussion

In our study in Ethiopia, we found that holy water priest healers hold diverse explanatory models or causal explanations on mental illness, and they were eclectic in their treatment approaches, emphasizing the religious and spiritual. The priest healers were generally welcoming and positive toward the cross-system collaboration, appreciated having a common goal in improving patient care, and many made particular effort to overcome family and patient reluctance to use the biomedical clinic. The priest healers particularly appreciated the respect shown by the biomedical practitioners and the recognition of their role and contribution in the mental health system. They highlighted the clinic's effectiveness in treating violent and aggressive patients, but there were notable concerns regarding the one-way only referrals from priests to the clinic, the lack of communication after referral, side effects of biomedical treatment, and loss of fundamental attention to the spiritual and meaning realm of care.

As the idiomatic title of our study suggests, the religious healers’ views on causes and approaches to mental illness are inclusive and practical. Although the most ubiquitous explanations were religious and spiritual, the diverse range of etiological understanding was also remarkable. It would be helpful in future research to examine more systematically and flesh out more fully such an eclectic explanatory model that explored the root causes of spiritual possessions, multiple mechanisms and pathways that explained the symptoms, treatments approaches and factors associated with prognosis and well-being. Historically, studies in Nigeria and Ethiopia, for example, have shown a high prevalence of spiritual causal beliefs (Erinosho & Ayorinde, 1978; Odejide, 1979; Prince, 1975). Research has also shown that generally the more religious people were, the more they tended to make spiritual attributions in their understanding of mental illness (Bhui et al., 2002). However, traditional healers’ evolving and increasingly mixed causal attributions that include biopsychosocial aspects have become evident in later studies. For instance, in a more recent Kenyan study, traditional healers cited a large range of biopsychosocial causes akin to our current findings (Mbwayo et al., 2013). Similarly, a study that reviewed the causal beliefs of patients attending traditional healers in Nigeria found the majority of respondents attributed their problem to psychosocial causes and fewer than half of them evoked spiritual causes (Ilechukwu, 1988). Moreover, a South African study found patients with severe mental illness attending traditional healers disliked their “bewitchment” diagnosis for its frightening and negative implications, and preferred multifactorial explanations (Robertson, 2006). More recent community surveys in Ethiopia show that psychosocial factors such as stress, drug abuse and poverty are increasingly being recognized as important etiological risk factors for mental illness (Deribew & Tamirat, 2005; Jacobsson & Merdasa, 1991; Mulatu, 1999), echoing similar findings from other global settings (Cohen et al., 2016).

Multifactorial etiological attribution is an encouraging finding in this study because it affirms the growing influence of a biopsychosocial model and provides empirical evidence against any assumption that the religious healers in this area believe solely in the spiritual etiologies of mental illnesses. Furthermore, the finding of religious healers’ inclusive and ecumenical etiological understanding constitutes a promising common ground for collaborative work (Good, 1986). Ironically, it is a worthwhile reflection that it is often the biomedical practitioners who are relatively more rigid in their biomedical theoretical silos with an “us and them” concept, often neglecting the psychosocial and spiritual realms of human experiences (Odebiyi, 1990; Offiong, 1999; Solera-Deuchar et al., 2020).

Contrary to common conceptions, the current study found that many religious healers were also eclectic and inclusive of non-traditional methods in their treatment approaches (Biswal et al., 2017). Many even employed biblical, philosophical and historical justifications for entering the collaboration, and persuading patients and family to use the additional biomedical modalities. This welcoming receptivity on the part of the religious healers may reflect the level of conviction they have about the Orthodox Church-based teaching, and the confidence they have on their professional identity. This is an encouraging finding from a global mental health perspective, and echoes some similar findings from Kenya and South Africa (Musyimi et al., 2016; Van Niekerk et al., 2014). It is also of note that productive collaboration may not necessarily depend on having completely shared etiological understanding. There may be more motivational reasons—such as putting the interest of patients first, and more conceptual overlaps between the two sectors than one imagines (Kleinman, 1978).

Our study also highlights the fact that the true challenge to achieving collaboration may be related less to the theoretical divide, and more to attitude and ways of interaction. The religious healers emphasized the importance of trust and respect as a key motivator for collaboration. Research has shown that trust is a critical prerequisite for successful collaboration between different systems of healthcare (Brown & Calnan, 2016; Gilson, 2003; Illingworth, 2002). What is less discussed, but is also essential, seems to be the topic of respect. A Kenyan study on the prospect of collaboration highlighted the gulf between biomedical practitioners and faith/traditional healers based on the latter's perceived and actual experiences of being slighted and devalued—often reported as “dirty” herbal healers by the former (Musyimi et al., 2016). Similarly, a South African study showed traditional healers felt that western physicians did not treat them with respect and undervalued their contribution to the health of the community (Sorsdahl et al., 2010a, 2010b). These issues are exemplified in the current study in mixed ways—on the one hand, participants have specifically indicated that the reason they favored the collaboration was based on feeling being respected; on the other hand, they experienced disrespect when the clinic turned away a “dirty” patient who was covered in holy mud, and when they received no two-way referrals. We learn here that trust and respect are underappreciated linchpins in collaborative work, and we gain insight into how traditional healing itself may be rooted in sociocultural principles of respect and reciprocity (Bierlich, 2007; Cocks & Moller, 2002; Krah, 2014; Sorsdahl et al., 2013).

Research has shown that traditional healers are often effective in treating patients suffering from less-critical mental disorders through counseling, and psychosocial and spiritual support (Ae-Ngibise et al., 2010; Campbell-Hall et al., 2010; Csordas, 1988; Kahn & Kelly, 2001; Krippner, 2012; Teuton et al., 2007). Many patients also preferentially choose religious healers over others, and often report greater improvement when compared with biomedicine or primary healthcare clinics (Kleinman & Sung, 1979; Lee et al., 2010; Patel et al., 1998), or psychotherapists (Koss, 1987). Our findings show that the religious healers know they are good at a wide range of mental health problems, including “grief, marital problem, family conflict, fear, hopelessness, and emotional and spiritual pain”; and what they are less comfortable in treating, in terms of acutely ill, agitated and aggressive patients. Although we did not inquire about the reasons behind these issues, we may surmise that—in addition to the respect and trust they hold, and the unconditional compassion and empathy they have talked about—they may be effective and offer more therapeutic power to restore meaning, purpose and hope in the lives of patients through the use of Scripture for believers (Wirth, 1995). Research has shown an association between religious involvement and improved mental health outcomes, and religiosity is potentially a strong protective factor against addiction (Edlund et al., 2010; Van der Meer Sanchez et al., 2008) and suicidal crises (Svob et al., 2018), among others.

Furthermore, the current study has indirectly underlined how culturally accepted, and less-stigmatized religious healing is compared with biomedical treatment (Corrigan et al., 2018; Mannarini & Rossi, 2018). Religious healers are known to focus on spiritual and social balance and harmony over personal psychopathology, use reframing to make sense of illness, work with family and social structures, and restore community identities and social cohesion, all aspects that are less-stigmatizing than focusing on personal psychopathology (Boelens et al., 2012; Kaptchuk, 2002; Kirmayer, 2004; Lemelson, 2004; Mainguy & Mehl-Madrona, 2013; Waldram, 2013). Improving mental health awareness, strengthening mental health literacy, and combating stigma may be some of the key areas in which biomedicine and traditional healers could find synergies and have a positive impact through collaborations (Egbe, 2015; Ganasen et al., 2008; Kitchener & Jorm, 2002; Rosen, 2003; Sewilam et al., 2015).

One interesting finding of the study is how the religious healers saw the biomedical clinic's ability to manage aggressive and violent patients. This recognition is also reported in similar studies elsewhere (Halliburton, 2004; Salib & Youakim, 2001). A recent systematic review found “little evidence to suggest that [traditional healers] change the course of severe mental illnesses” (Nortje et al., 2016). In reality, many severely mentally ill patients do seek, or are brought to religious healers and are managed by restraints (often chained) (Abbo et al., 2012; Fekadu & Thornicroft, 2014; Hanlon et al., 2010; Harding, 1973; Sorketti et al., 2013) The perceived and known efficacy of biomedicine may provide an opportunity or platform to further engage traditional and religious healers for productive collaboration, in which one could combine the advantages of biomedicine and religious healers’ effective psychosocial interventions.

Last but not least, our study found that religious healers held very strong views that spiritual care should be at the core of treating mental illness, and that it was missing from biomedical care. To that end, our finding is in keeping with research that found traditional healers perceive western care as being useful for temporary measures or as a last resort, and consider that religious treatment is, broadly speaking, better (Sorsdahl et al., 2010a, 2010b). This is a very sobering and timely reminder of the perceived over-medicalization of mental illness in western medicine (Huda, 2021). Many western psychotherapies have been proponents of this deeper personal level of healing, but patients still miss a range of larger community and spiritual involvement. With the right frame of mind, biomedicine could potentially benefit from transfers of knowledge and practices from spiritual healing, and improve our overall holistic care (Koch & Binder, 2013).

## Limitations

The sample of religious healers came from two large, popular, urban, Orthodox churches, and this limits the representativeness and generalizability of the findings. The convenience sampling approach, using participants identified by church leaders, may potentially introduce sampling bias in an over-representation of priests who are cooperative and favorable to the project. We have made efforts to encourage objectivity, promote personal views and maintain anonymity through our research framework and methodology to ameliorate this potential bias.

## Conclusion

The current qualitative study of a rare cross-system collaboration in a Developing World setting shows promising receptivity toward collaboration, gains knowledge on the spiritual focus and empathic power of the traditional healers, and identifies humility and mutual respect as underappreciated key ingredients for success. Future attempts on improving and expanding the collaborative approach to scale-up mental healthcare should pay close attention to these issues.
